# Using an Image Fusion Methodology to Improve Efficiency and Traceability of Posterior Pole Vessel Analysis by ROPtool

**DOI:** 10.2174/1874364101711010143

**Published:** 2017-06-29

**Authors:** Sasapin.G. Prakalapakorn, Laura A. Vickers, Rolando Estrada, Sharon F. Freedman, Carlo Tomasi, Sina Farsiu, David K. Wallace

**Affiliations:** 1 *Deptartment of Ophthalmology, Duke University, Durham, NC 27710, USA*; 2 *Deptartment of Computer Science, Duke University, Durham, NC 27708, USA*; 3 *Deptartment of Biomedical Engineering, Duke University, Durham, NC 27708, USA*

**Keywords:** Image analysis, Retinopathy of prematurity, ROPtool, Vessel analysis

## Abstract

**Background:**

The diagnosis of plus disease in retinopathy of prematurity (ROP) largely determines the need for treatment; however, this diagnosis is subjective. To make the diagnosis of plus disease more objective, semi-automated computer programs (*e.g.* ROPtool) have been created to quantify vascular dilation and tortuosity. ROPtool can accurately analyze blood vessels only in images with very good quality, but many still images captured by indirect ophthalmoscopy have insufficient image quality for ROPtool analysis.

**Purpose:**

To evaluate the ability of an image fusion methodology (robust mosaicing) to increase the efficiency and traceability of posterior pole vessel analysis by ROPtool.

**Materials and Methodology:**

We retrospectively reviewed video indirect ophthalmoscopy images acquired during routine ROP examinations and selected the best unenhanced still image from the video for each infant. Robust mosaicing was used to create an enhanced mosaic image from the same video for each eye. We evaluated the time required for ROPtool analysis as well as ROPtool’s ability to analyze vessels in enhanced vs. unenhanced images.

**Results:**

We included 39 eyes of 39 infants. ROPtool analysis was faster (125 vs. 152 seconds; *p*=0.02) in enhanced vs. unenhanced images, respectively. ROPtool was able to trace retinal vessels in more quadrants (143/156, 92% vs 115/156, 74%; p=0.16) in enhanced mosaic vs. unenhanced still images, respectively and in more overall (38/39, 97% vs. 34/39, 87%; p=0.07) enhanced mosaic vs. unenhanced still images, respectively.

**Conclusion:**

Retinal image enhancement using robust mosaicing advances efforts to automate grading of posterior pole disease in ROP.

## INTRODUCTION

In retinopathy of prematurity (ROP), the presence of plus disease largely determines if treatment is needed [1]. However, the diagnosis of plus disease is subjective and, even among ophthalmologists who are experienced in ROP screening, agreement of plus disease diagnosis is poor [2, 3]. Thus, there is a need to make the diagnosis of plus disease more objective.

Multiple computer programs have been developed to quantify vascular characteristics such as tortuosity and/or dilation [4-7]. ROPtool is a semi-automated computer program that analyzes vascular tortuosity and dilation in retinal images [5, 8, 9]. In analysis of high-quality Retcam images, ROPtool has been shown to have high sensitivity and specificity for diagnosis of plus disease when compared to masked examiners experienced in ROP diagnosis [8]. While ROPtool can accurately detect characteristics suggestive of plus disease, a previous study found that ROPtool could trace vessels in only 43% of quadrants in video indirect ophthalmoscopy (VIO) still images due to insufficient image quality [10].

A previous pilot study demonstrated that an image fusion methodology (robust mosaicing) could generate enhanced images from VIO videos [11]. The process of robust mosaicing converts a VIO recording into a single, high-quality, enhanced mosaic image with a wider field-of-view than a single still image created from the same video recording. This enhanced image is created by minimizing image artifact, enhancing blood vessels, and blending the best video image frames [11]. In the current study, we systematically evaluated the ability of robust mosaicing to increase the efficiency and image traceability of posterior pole vessel analysis by ROPtool.

## MATERIALS AND METHODOLOGY

This study was approved by the Duke Health System Institutional Review Board and was in compliance with regulations of the United States Health Insurance Portability and Accountability Act and the Declaration of Helsinki. This was a retrospective study of infants enrolled in a previous prospective study looking at the use of ROPtool to diagnose plus disease in real-time at the bedside during ROP rounds [12]. In the previous study, all infants in the Duke Hospital neonatal intensive care unit (NICU) who were screened for ROP between September 2010-March 2011 were eligible for inclusion. All ROP examinations were performed by one of two ophthalmologists experienced in ROP screening (SFF and DKW). In our NICU, we obtain VIO images on all infants during routine ROP screening rounds. As part of the previous study, infants had quadrant-level grading for vessel dilation and tortuosity during their routine clinical examination. For each quadrant, vessel dilation and tortuosity were graded as follows: for dilation, a score of 0 = thin, 1 = normal, 2 = pre-plus and 3 = plus; for tortuosity, 0 = straight, 1 = normal, 2 = pre-plus and 3 = splus.

For this study, infants were eligible for inclusion if they had VIO images and quadrant-level grading for vessel dilation and tortuosity obtained during their clinical examination. For each eligible infant, a single examination date was chosen for inclusion in our study. To enhance our sample with abnormal posterior pole findings, we chose the examination date with the most severe posterior pole disease (plus > pre-plus > normal) and that was closest to the infant’s post-menstrual age (PMA) of 36 weeks. If there were two such examinations equidistant to 36 weeks PMA, the later examination was selected. Only right eyes were included. Infants included in a previous pilot study were excluded [11]. Eyes with prior laser treatment were excluded.

For the selected examination date for each infant, two different still images were created: **1)** an unenhanced still image and **2)** an enhanced mosaic image. The unenhanced still image for each infant was created by having one of the authors (LAV) review the VIO video and manually select the single best still frame with regard to centration on the optic nerve and image quality using MovieMaker v.1.1.2427.0 (Microsoft Corporation, Redmond, WA). Image quality was based on focus and number of blood vessels visible. The enhanced mosaic images were created by processing the VIO video with the mosaicing software program [11]. The mosaicing program works as follows: The user uploads the VIO file into the program. The program selects a still image from the VIO file and presents it to the user. The user then selects four points on the optic nerve border and four random retinal points to indicate the color of the retina in a particular video. Then, the program processes the video file and selects a series of still images to create and output a single enhanced mosaic image.

**Fig. (1):**
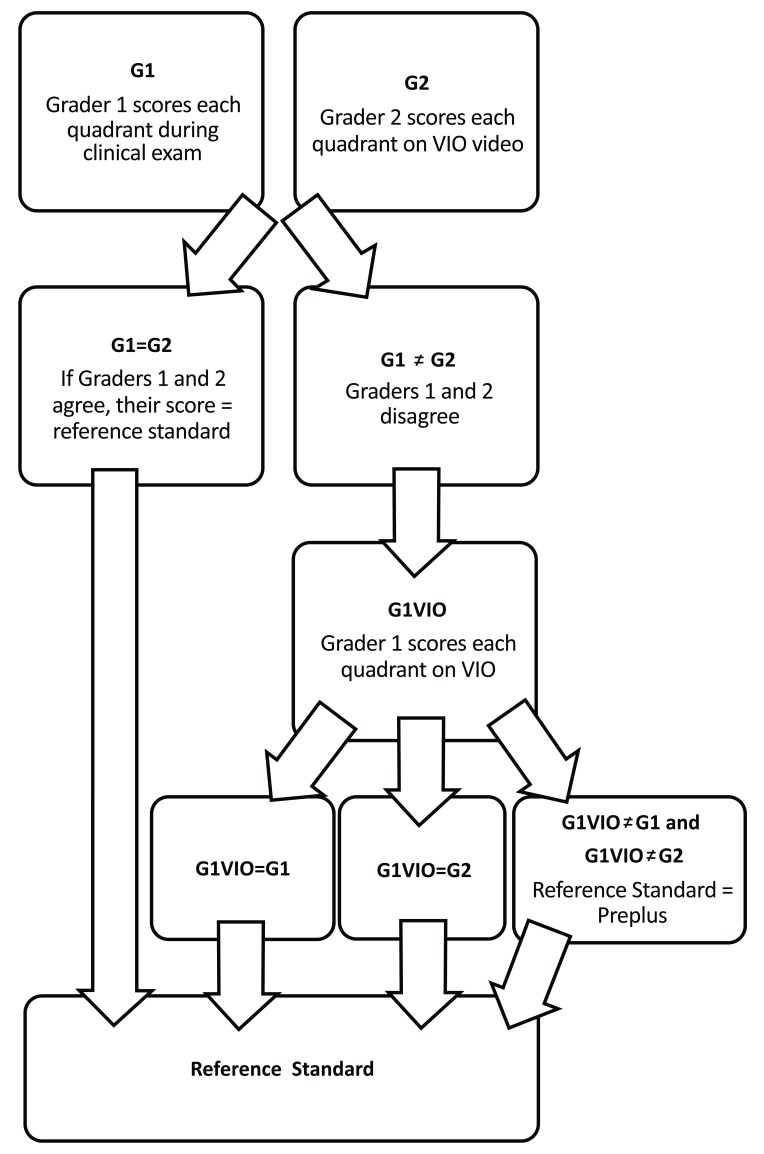
Algorithm for establishing the reference standard.

A “reference standard” diagnosis for the presence of posterior pole disease was established for each infant to determine the accuracy of posterior pole vessel analysis with ROPtool. For this study, the reference standard was established using a combination of the clinical examination findings and scoring of the VIO images taken from the same examination. This was carried out in the following manner (Fig. **1**).

First, we determined which examiner (DKW or SFF) performed the clinical examination and quadrant-level grading on the infant on the selected examination date and then we had the other examiner (DWK or SFF) review the VIO video from that same examination session and score each quadrant using the same quadrant-level scoring system described above for dilation and tortuosity. Then we placed each quadrant into one of three categories (plus, pre-plus, or neither) based on its dilation and tortuosity scores. A quadrant was considered “Plus” if the quadrant had a score of 3 for tortuosity and 3 for dilation (plus-level tortuosity and dilation, respectively); “Pre-plus” if the quadrant had a score of 2 for dilation and 2 for tortuosity (pre-plus level dilation and tortuosity, respectively), 2 for dilation and 3 for tortuosity, or 3 for dilation and 2 for tortuosity; or “Neither” if the quadrant had any other combination of dilation and tortuosity scores. We then compared the initial quadrant-level clinical examination score to the score given by the other examiner when evaluating the VIO video. If these two scores were in agreement on the diagnosis as being either plus, pre-plus, or neither then this became the reference standard. If they were not in agreement, then the first grader, who had performed the clinical examination, was also asked to review the VIO video from the same examination session and score each quadrant. If two of these three quadrant-level scores were in agreement, that score became the reference standard. If all three scores were in disagreement (one with a diagnosis of plus, one with pre-plus, and one with neither) for a given quadrant, then the quadrant was categorized as pre-plus.

Eye-level analysis was also performed by categorizing eyes into one of 3 groups: Plus, pre-plus, or neither. We considered an eye “plus” if at least two quadrants were scored as plus; “pre-plus” if at least two quadrants were scored pre-plus and the eye did not fulfill criteria to be categorized as plus; or “neither” if it did not fall into either of the previous two categories.

### ROPtool Analysis of Enhanced and Unenhanced Images

We used ROPtool v2.1.8 to analyze both the unenhanced still and the enhanced mosaic images in a randomized order. ROPtool works as follows: The user uploads an image into ROPtool. The user identifies the optic nerve (by choosing four cardinal points on the optic nerve border) and the center of the macula. While ROPtool then delineates four quadrants, the user can modify the areas chosen for each quadrant. For this study, whenever possible, the user ensured that ≥ 2 major vessels were included in each quadrant, typically the most prominent arteriole and venule. We recorded the ability of ROPtool to trace the vessels in each quadrant (traceability), and the time required for ROPtool analysis (which includes the time for the user to run ROPtool). A “traceable” quadrant was defined as having ≥ 2 vessels that could each be traced for at least one disc diameter from the optic nerve. A “traceable” image was defined as having ≥ 2 traceable quadrants. One of the authors (LAV) performed all ROPtool analysis.

The results of the ROPtool analysis for the manually selected best unenhanced still images and the enhanced mosaic images were then compared to our reference standard. We investigated the ROPtool indices of tortuosity weighted plus (TWP) and sum of adjusted indices (SAI). TWP is a parameter that gives more weight to dilation as tortuosity increases. SAI is a parameter that assigns equal weight to dilation and tortuosity. We created receiver operating characteristic (ROC) curves for TWP and SAI compared to the reference standard. Then we compared the area under the curve (AUC) for the ROC curves of the unenhanced still images and the enhanced mosaic images. The correlation between the four quadrants within each eye per infant was accounted for using generalized estimating equations to compare the AUC’s and correlated McNemar’s test to compare proportion of traceable quadrants between the unenhanced still and the enhanced mosaic images [13].

All statistical analysis was performed with SAS v.9.1.3 (SAS Institute Inc, Cary, NC) and JMP v.10.0.0 (SAS Institute Inc, Cary, NC).

## RESULTS

### Description of Cohort

Of the 62 infants included in the previous study [12], 39 infants fulfilled inclusion criteria for this study. Twenty-three infants were excluded from this study because they were included in a prior study (N=15) [11], had prior laser treatment (N=3), or did not have an available VIO recording or quadrant-level grading (N=5). Of the infants included in this study, the mean gestational age was 27 weeks (range: 24-35), mean birth weight was 885 grams (range: 540-1660), and mean PMA was 36 weeks (range: 32-49).

Of the 156 quadrants available for analysis, using our algorithm to establish the “reference standard” diagnosis for posterior pole disease, 10 quadrants (6%) had plus disease, 20 quadrants (13%) pre-plus disease, and 126 quadrants (81%) had neither. There were 8/156 (5%) quadrants where the three clinical scores were in disagreement, and these quadrants were categorized as pre-plus. On eye-level analysis, 4 eyes (10%) had plus disease, 4 eyes (10%) had pre-plus disease, and 31 eyes (80%) had neither.

### Traceability of Images

On the quadrant-level, ROPtool was able to trace more quadrants in the enhanced mosaic (143/156, 92%) vs. unenhanced still (115/156, 74%) images Fig. (**2**). When the four quadrants within each eye per infant were accounted for, this difference was not statistically significant (*p=*0.16). On the eye-level, more enhanced mosaic (38/39, 97%) vs. unenhanced still (34/39, 87%) images were traceable (*p*=0.07). Average time for ROPtool analysis of an entire image was faster (125 vs. 152 seconds; *p* =0.02) for enhanced mosaic vs. unenhanced still images, respectively.

### Accuracy of Diagnosing Plus Disease

**Fig. (3):**
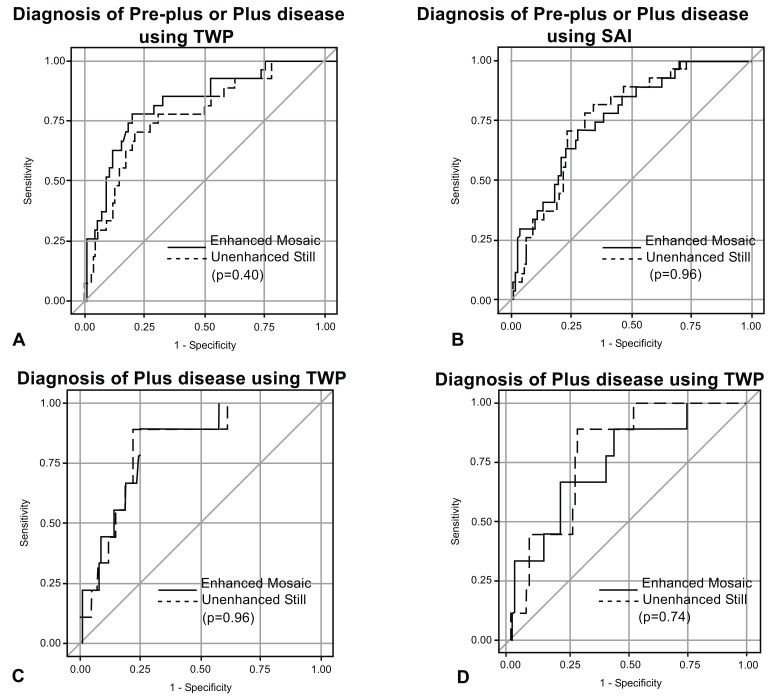
Receiver operating characteristic (ROC) curves comparing unenhanced still and enhanced mosaic images. Diagnosis of pre-plus or plus disease (A and B) or plus disease (C and D) using tortuosity weighted plus (TWP) (A and C) and sum of adjusted indices (SAI) (B and D). AUC, area under the curve. p-values are given comparing the differences between the AUC’s.

The AUC’s for detection of plus disease alone or of pre-plus or plus disease was compared between the enhanced mosaics and unenhanced stills. There was no statistically significant difference between them for the indices of TWP or SAI (p>0.05) (Table **1**, Fig. **3**).

**Fig. (4):**
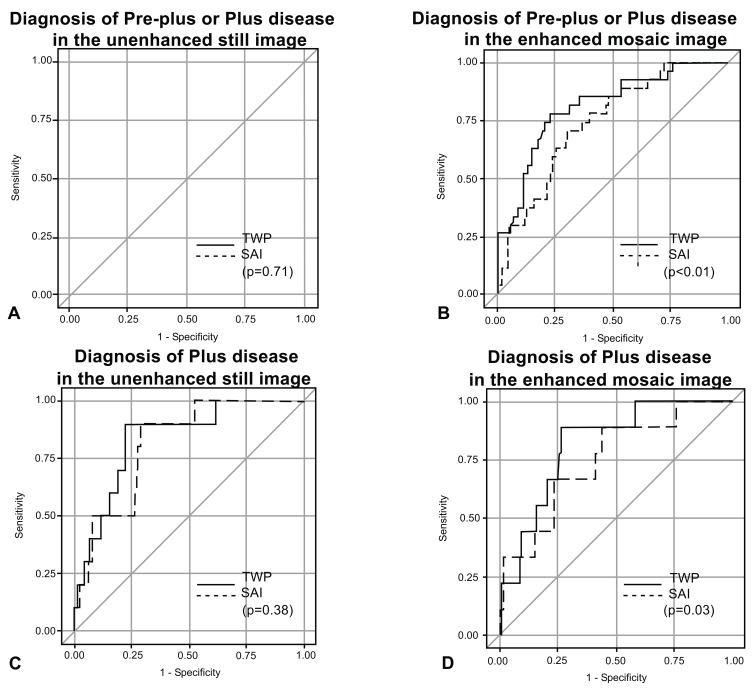
Receiver operating characteristic (ROC) curves comparing the indices of tortuosity weighted plus (TWP) and sum of adjusted indices (SAI) to diagnose pre-plus or plus disease (A and B) or plus disease (C and D). ROC curves for the unenhanced still images (A and C) and the corresponding enhanced mosaic images (B and D). AUC, area under the curve. p-values are given comparing the differences between the AUC’s.

The AUC’s for detection of plus disease alone or of pre-plus or plus disease was compared for the indices of TWP and SAI (Table **1**, Fig.**4**). For the unenhanced still images, AUC for TWP was greater than SAI for both outcomes, although not statistically significant in either scenario Figs. (**4A** and **C**). For the enhanced mosaic images, there was a statistically significant difference for the AUC for TWP vs. SAI for both outcomes Fig. (**4B** and **D**).

## DISCUSSION

The process of robust mosaicing can create enhanced mosaic images from VIO video images, leading to improved efficiency and traceability of posterior pole blood vessel analysis by ROPtool. We found that compared to using a manually selected best still image from a VIO video, an enhanced mosaic image decreased the time required for ROPtool to analyze posterior pole vessels by 18% (an average of 27 seconds/image; (*p* =0.02) and increased the ability of ROPtool to trace still images by 10% (p*=*0.07).

Analysis of images processed by robust mosaicing (*i.e.* enhanced mosaic images) has multiple advantages to analysis of manually selected best still images. A prior study suggested that vascular pathology may not be well represented by one frame from the VIO recording [10]. Robust mosaicing addresses this limitation by creating an enhanced mosaic image with a wider field-of-view compared to a manually selected still image from the same VIO video file, so that more data is captured within the enhanced image. This previous study also found that darker fundus pigmentation was associated with a decreased ability of ROPtool to analyze images, probably due to a decrease in contrast between the blood vessels and the retina [10]. Robust mosaicing also addresses this limitation by removing lens and compression artifact, minimizing color distortion, and enhancing vessels, all of which increases contrast between vascular and non-vascular structures to facilitate tracing vessels by ROPtool. The current study demonstrates that robust mosaicing decreases the time required to trace vessels using ROPtool, and increases the number of vessels, quadrants, and images that are traceable by ROPtool. While we did not systematically record the time it took to generate an enhanced mosaic image with the mosaic program or to manually select the best unenhanced still image from the VIO file, we noted that the time required to generate an enhanced mosaic image (including the time for the user to run the mosaic program and the time for the program to process the image) was approximately 2 minutes, while the time required to manually select the best unenhanced still image from the VIO file was approximately 2.5 minutes. Thus, when considering total time from image selection through ROPtool analysis, the mosaic program appears to improve efficiency.

While robust mosaicing increased the efficiency and traceability of vessels in posterior pole disease analysis by ROPtool, we did not find a change in ROPtool’s ability to accurately diagnose posterior pole disease using the enhanced mosaics compared to the unenhanced stills. A prior study identified the ROPtool indices of TWP and SAI as having the highest overall accuracy for diagnosing plus disease compared to other ROPtool indices [14]. We found that the overall diagnostic performance (using AUC) in enhanced mosaic vs. unenhanced still images were similar for TWP and SAI.

Our study had several limitations. Because we included only images obtained from September 2010-March 2011, our sample size was relatively small. The manual selection of a single best frame from the video is subjective and will likely differ between individuals. Also, the mosaicing program is semi-automated and requires the user to select points on the optic nerve border and retina; therefore, depending on user input, the resulting enhanced mosaic image may differ between users. In addition, the ROPtool program is also semi-automated and depends on user input in detecting the optic nerve head border and center of the macula, also introducing inter-user variability. Although it has been shown that there is high interuser agreement (95%) between ROP experts using ROPtool for the determination of tortuosity sufficient for plus disease [15], the extent or importance of variability in vessel analysis has not been evaluated for the either the mosaicing program or ROPtool between less experienced users.

## CONCLUSION

Retinal image enhancement using robust mosaicing advances efforts to automate the grading of posterior pole disease in ROP by making computer programs that analyze vascular characteristics (*e.g.* ROPtool) more efficient. Automation of plus disease diagnosis could make ROP screening more objective. The increasing demand for ROP screening around the world and the limited access to ROP experts in some areas highlights the importance of increasing the automation, efficiency, and objectivity of ROP examination.

## Figures and Tables

**Fig. (2):**
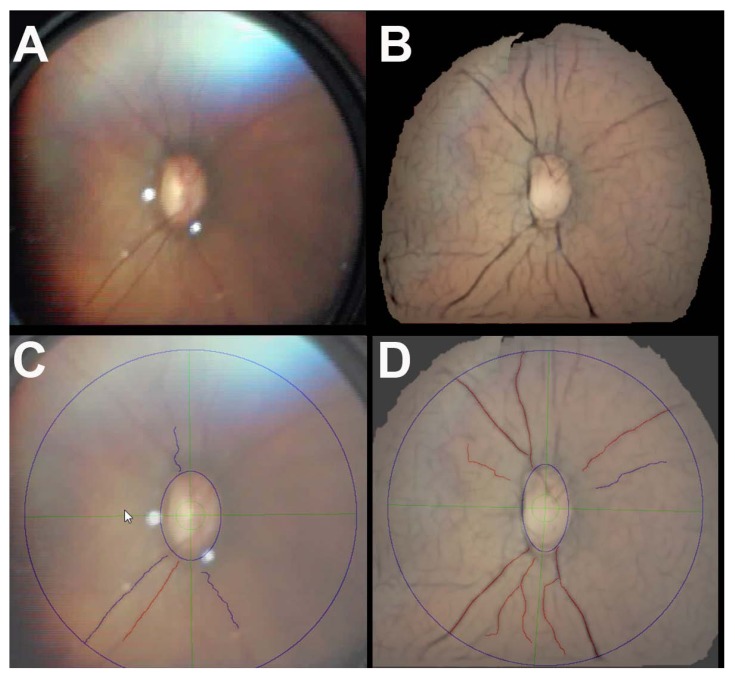
Retinal images of a premature infant’s eye, acquired using video indirect ophthalmoscopy (VIO), and the corresponding ROPtool tracing of the major blood vessels in each of these images. A quadrant was considered “traceable” by ROPtool if it had at least two vessels that could each be traced for at least one disc diameter from the optic nerve. A) Best unenhanced still image selected from the VIO video. B) The corresponding enhanced mosaic image created using an image fusion methodology to minimize artifact, enhance contrast, and increase field-of-view. C) ROPtool tracing of the unenhanced still image (shown in A) with only one quadrant traceable by ROPtool. D) ROPtool tracing of the corresponding enhanced mosaic image (shown in B) with all four quadrants traceable by ROPtool.

**Table 1:** Area under the curve (AUC) for receiver operating characteristic curves with predictors of pre-plus or plus disease, or plus disease only.

**Outcome Variable**	**Parameter**	**Image**	**AUC**
Pre-plus or Plus Disease	TWP	Enhanced mosaic	0.80
	TWP	Unenhanced still	0.78
	SAI	Enhanced mosaic	0.74
	SAI	Unenhanced still	0.77
Plus Disease Only	TWP	Enhanced mosaic	0.81
	TWP	Unenhanced still	0.84
	SAI	Enhanced mosaic	0.74
	SAI	Unenhanced still	0.82

TWP, tortuosity weighted plus; SAI, sum of adjusted indices.
